# Lymph-node ratio is an independent prognostic factor in patients with stage III colorectal cancer: a retrospective study from the Middle East

**DOI:** 10.1186/1477-7819-10-63

**Published:** 2012-04-25

**Authors:** Elias Elias, Deborah Mukherji, Walid Faraj, Mohammad Khalife, Hani Dimassi, Mohamad Eloubeidi, Hasan Hattoum, Ghassan K Abou‒Alfa, Ahmad Saleh, Ali Shamseddine

**Affiliations:** 1 Division of Hematology and Oncology, Department of Internal Medicine, American University of Beirut, Riad El-Solh, Beirut, 1107 2020, Lebanon; 2 Division of Hepatobiliary and Gastric surgery, Department of General Surgery, American University of Beirut, Riad El-Solh, Beirut, 1107 2020, Lebanon; 3 School of Pharmacy, Lebanese American University, Byblos, Lebanon; 4 Division of Gastroenterology, Department of Internal Medicine, American University of Beirut, Riad El-Solh, Beirut, 1107 2020, Lebanon; 5 Department of Internal Medicine, Staten Island University Hospital, 475 Seaview Ave, Staten Island, NY, 10305, USA; 6 Memorial Sloan-Kettering Cancer Center, Section of Gastrointestinal Oncology, New York, NY, USA

**Keywords:** Colorectal cancer, Stage III, Lymph node ratio, Prognosis

## Abstract

**Background:**

In this retrospective study, we evaluated the prognostic effect of positive lymph-node ratio (pLNR) on patients with stage III colorectal cancer (CRC). Our paper is the first analysis, to our knowledge, to deal with such data from the Middle East.

**Methods:**

We analyzed the clinicopathological data of 535 patients diagnosed with colorectal cancer at our institution between 1983 and 2003. The 164 patients diagnosed with stage III disease were divided into two categories based on lymph-node ratio (LNR) being the ratio of positive lymph nodes over total lymph nodes dissected: LNR ≤0.4 and LNR >0.4. We used Kaplan-Meier and Cox proportional hazard models to evaluate the prognostic effect of pLNR.

**Results:**

The 10-year survival rate for the patients with stage IIIA, IIIB and IIIC cancers were 76%, 56% and 0% respectively (*P* = 0.014). Using pLNR of 0.4 as the cutoff point was found to yield clinically and significant results, with a significant difference in the outcomes of patients with pLNR ≤0.4 compared to those with pLNR >0.4 (hazard ratio = 5.25, 95% confidence interval = 1.2 to 22.1, *P* = 0.02).

**Conclusion:**

The ratio-based staging (pLNR) of CRC is a more accurate and clinically useful prognostic method than the number of positive LNs resected or the total number of LNs retrieved for predicting the course of patients with stage III CRC.

## Background

Colorectal cancer (CRC) is the third most common cancer in both female and male populations [1]. Currently, its progression is staged using the TNM (tumor, node, metastasis) staging system according to tumor size, lymph-node involvement and distant metastases, as recommended by the American Joint Committee on Cancer (AJCC) [2]. However, many investigators have questioned the prognostic power of the TNM system because of the possibility of stage migration, and have proposed alternative prognostic methods.

One notable alternative bases a patient’s prognosis upon the total number of LNs resected. This concept has long been debated in the literature. Many studies have shown that a higher number of LNs retrieved leads to more accurate staging and apparently improved survival outcomes [3-8]. Furthermore, a study conducted by the National Cancer Institute (NCI) involving 60,000 patients illustrated a relationship between the number of resected positive LNs and the survival rate in stage III patients [9]. This relationship has driven studies that have attemped to set recommendations for the number of LNs that should be resected. The NCI and the Royal College of Pathologists (RCP) agree on the recommendation for a minimum of 12 LNs to be resected [10].

Some researchers have also investigated the importance of the number of negative LNs retrieved on the survival outcome [11], but further studies are required in this area.

Methods such as using the positive LN ratio (pLNR; the number of positive LNs divided by the total number of LNs resected) have been reported as significant prognostic factors in malignancies of the pancreas, stomach, bladder, breast and esophagus [12-15], but the importance of the pLNR as a prognostic factor in colon cancer is still being explored.

In this retrospective study, we aimed to evaluate the importance of pLNR as a prognostic parameter on the survival of patients diagnosed with stage III colon cancer in our population, and to compare its prognostic power against other methods, such as the total number of LNs and the number of positive LNs resected. To our knowledge, it is the first study to deal with this topic in the Middle East, hence we relied on the world literature for purposes of comparison and references.

## Methods

The study enrolled 535 patients who were diagnosed with CRC at our institution between 1983 and 2003. Of the 535 patients, 164 were diagnosed with stage III disease, which constituted the sample to be analyzed. The clinicopathological variables reviewed included age at presentation, gender, personal and family medical history, social habits, symptoms, sites of neoplasms, diagnostic tools, pathology results, grade, TNM staging, type of surgery undergone, adjuvant therapy, and survival rate.

We defined survival rate as overall survival from the time of diagnosis to either the time of death or the last follow-up. Tumor grade was classified as low-grade (well or moderately differentiated) and high-grade (poorly differentiated, anaplastic, or undifferentiated). Curative resection was defined as clear pathological margins after surgery, and the follow-up period was 10 years.

### Statistical analysis

Abstracted data from the medical records of the 535 patients were coded and analyzed using SPSS(software, version 18; SPSS Inc., Chicago, IL, USA). Summary statistics were computed for patients with stage III disease. Survival rates at 1, 5 and 10 years were computed using the Kaplan-Meier method, and the log rank test was used to calculate the *P*-values for the different variables. A multivariate model using the Cox proportionate hazard technique was created using age and gender of the participants as control variables, and all significant variables at the bivariate level, as well as important prognostic variables such as treatment and number of positive LNs. Coefficients and standard errors were exponentiated to create hazard ratios (HR) and 95% confidence intervals. *P* < 0.05 was considered significant.

## Results

### Clinicopathologic characteristics of the patients

Of the 164 patients diagnosed with stage III disease, 79 (48.2%) were male and 85 (51.8%) were female. The mean age at presentation was 57.05 years (range 12–97) (Table 1).

**Table 1:** Distribution of patients’ characteristics

	**N**	**%**
Age		
Mean	57.05	
Range	12 – 97	
SD	16.73	
Gender		
Male	79	48.2
Female	85	51.8
Total LNs resected		23.6
Mean	20.86	
Range	2 to 88	
SD	15.2	
< 12	38	
≥ 12	123	76.4
Grade		
Well-differentiated	6	4
Moderately differentiated	93	61.6
Poorly differentiated	52	34.4
N staging		
N1 (1 to 3 LNs)	96	60.4
N2 (4+ LNs)	63	39.6

LN, LNs.

Presenting symptoms varied, and included: change in bowel habits (92 patients; 56.1%), abdominal pain (85; 51.8%), bleeding per rectum (80; 48.8%), weight loss (78; 47.6%), anemia (20;12.2%) and abdominal mass (2; 1.2%).

There were 56 patients (34.1%) diagnosed with left colon tumors and 27 patients (16.4%) with right-sided cancers. Rectal cancer was present in 35 patients (21.3%) while the transverse colon was involved in 5 patients (3%). Data were missing for 41 patients (25%).

Colonoscopy was performed in 72 patients (45%), barium enema in 39 (24.4%), sigmoidoscopy in 17 (10.6%), and CT scan in 16 (10%). Other investigations included kidney, ureter, and bladder X-ray, and digital rectal examination, and diagnosis was an incidental findings for some patients.

### Grade and stage

The 164 stage III patients were further subdivided into IIIA (14; 8.5%), IIIB (108; 65.8%), and IIIC (42; 25.6%). Adenocarciomas were moderately differentiated in 93 patients (61.6%), poorly differentiated in 52 patients (34.4%) and well differentiated in 6 patients (4%).

### Treatment

Of the 164 patients, 159 (97%) underwent surgery as part of their treatment, with 26.8% undergoing low anterior resection and 25% undergoing right hemicolectomy. Only three patients did not undergo any surgery, and data were missing for 2 patients (1.2%).

Neoadjuvant chemotherapy was given to 8 patients (4.9%), adjuvant chemotherapy to 81 patients (49.4%) and radiotherapy to 44 patients (26.8%).

### LN and LNR

The number of resected LNs (mean ± standard deviation) was 20.86 ± 15.22 (range 2–88).

**Figure 1:**
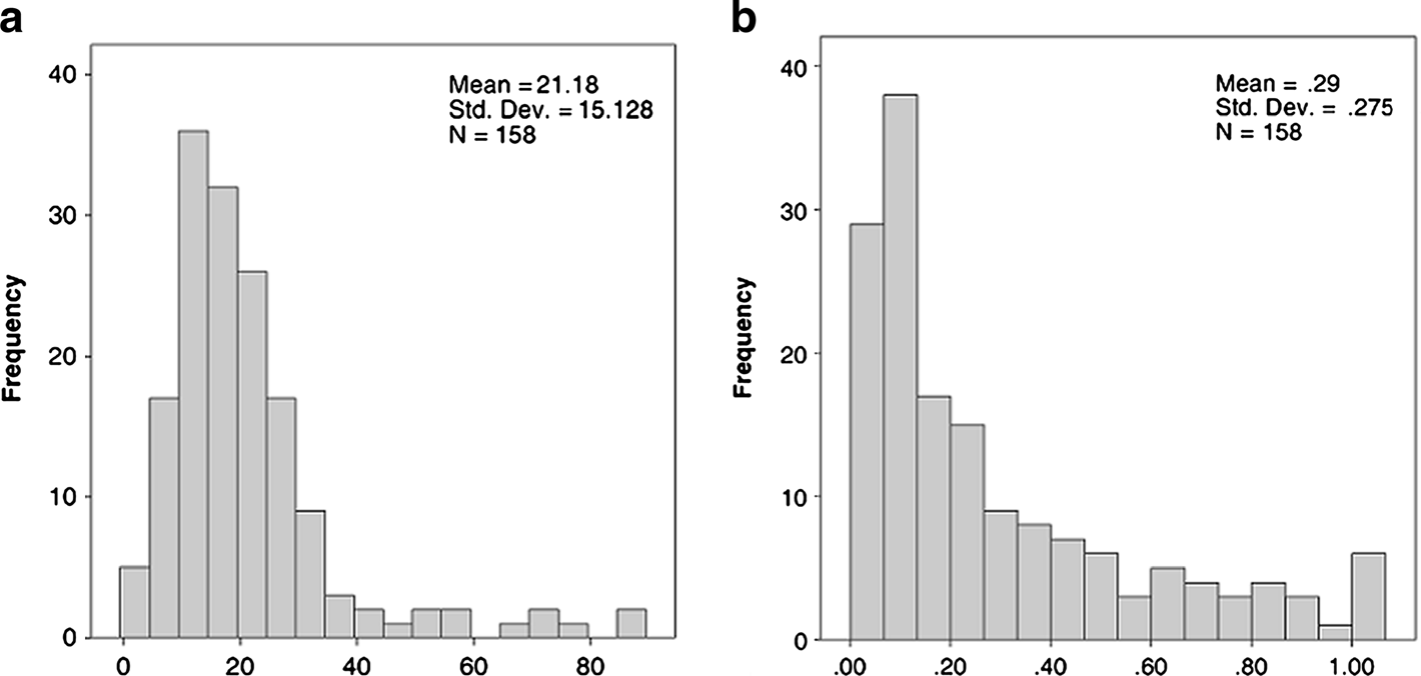
**Distribution of the number of dissected LNs (LNs). (a)** On average, 21 lymph nodes were dissected (median 18). The distribution of dissected LNs showed that most of the cases had between 2 and 40 LNs removed, with the distribution skewed positively to the right by some scattered cases with a higher number of dissected LNs. **(b)** Distribution of the ratio of positive to dissected LNs (lymph-node ratio; LNR). The average ratio of positive to dissected LNs was 0.29 (median 0.17), with 50% of the cases being between 0.01 and 0.17, and 75% of the cases between 0.01 and 0.42. There is a a positive skew to the right.

Based on the total number of LNs, patients were divided into two groups, those with fewer than 12 LNs resected (LN1 <12) and those with 12 or more nodes resected (LN2 ≥12); 38 patients (23.6%) had LN1 <12 and 123 (76.4%) had ≥12 (Table 1; Figure 1a).

We then divided our population according to the number of positive LNs retrieved, based on the AJCC classification; 96 patients (60.4%) were classified as N1 and 63 (39.6%) as N2.

We further divided the patients into two groups based on the LNR: 0 to ≤ 0.4 (116 patients; 74.8%) and > 0.4 (41; 25.2%) (Figure 1b).

Moreover, when stratifying our data according to positive LN and LNR we got the results as cited in Table 2.

**Table 2:** Cross tabulation of the variables according to LNR and positive LN

**Characteristics**	**LNR N (%)**		***P*-value**	**Positive LN**		***P*-value**
	**0 – 0.4**	**> 0.4**		**1 – 3**	**4+**	
**Age**	38 (69.1)	17 (30.9)	0.743	31 (55.4)	25 (44.6)	0.32
<50	25 (78.1)	7 (21.9)		17 (53.1)	15 (46.9)	
50 to 60	28 (77.8)	8 (22.2)		26 (72.2)	10 (27.8)	
61 to 70	26 (74.3)	9 (25.7)		22 (62.9)	13 (37.1)	
> 71						
**Stage III**						
IIIA	12 (100)	0 (0)	**< 0.001**	13 (100)	0 (0)	**< 0.01**
IIIB	95 (88)	13 (12)		81 (75)	27 (25)	
IIIC	10 (26.3)	28 (73.7)		2 (5.3)	36 (94.7)	
**Adjuvant chemotherapy**						
No	58 (71.6)	23 (28.4)	0.356	50 (61.7)	31 (38.3)	0.73
Yes	31 (79.5)	8 (20.5)		26 (65)	14 (35)	
**Gender**						
Female	65 (80.2)	16 (19.8)	0.068	44 (57.1)	33 (42.9)	0.42
Male	52 (67.5)	25 (32.5)		52 (63.4)	30 (36.6)	
**LNs resected**						
0 – 11	25 (71.4)	10 (28.6)	0.688	27 (77.1)	8 (22.9)	**0.02**
≥ 12	92 (74.8)	31 (25.2)		68 (55.3)	55 (44.7)	
**Grade**						
Well-differentiated	6 (100)	0 (0)	**0.002**	5 (83.3)	1 (16.7)	0.21
Moderately differentiated	75 (82.4)	16 (17.6)		58 (63)	34 (37)	
Poorly differentiated	29 (58)	21 (42)		26 (52)	24 (48)	
**Recurrence**						
No	23 (76.7)	7 (23.3)	0.923	16 (53.3)	14 (46.7)	0.11
Yes	50 (75.8)	16 (24.2)		47 (70.1)	20 (29.9)	

LNs, Lymph Nodes.

### Univariate analysis

**Figure 2:**
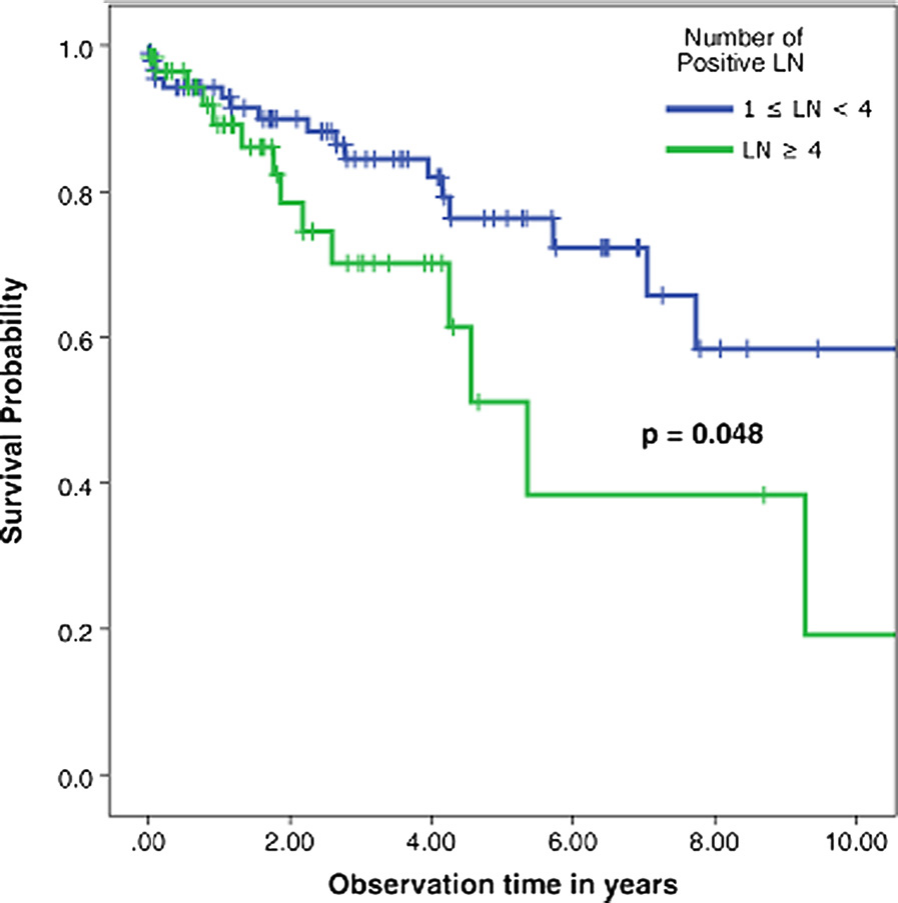
Survival curve for node-positive patients with colorectal cancer stratified by N stage (N1 = 1 to 3 positive LNs; N2 = > 3 positive LNs).

Stratifying stage III patients alone, survival at 1, 5 and 10 years, respectively, was 91%, 75.8% and 75.8% for stage IIIA; 94%, 77.1% and 56% for stage IIIB, and 84.7%, 22% and 0% for stage IIIC respectively (Table 3, Figure 2).

**Table 3:** Univariate survival analysis of the patients with stage III colorectal cancer

**Characteristics**	**N**	**1 year survival (%)**	**5 year survival (%)**	**10 year survival (%)**	***P* value**
**Age**					
<50	56	95.6 ± 2.9	79.1 ± 6.7	70.3 ± 10.2	
50 to 60	33	100	64.3 ± 13.2	42.9 ± 19.5	
61 to 70	35	90 ± 5.5	70.1 ± 11.2	19.5 ± 16.6	
> 71	39	80.5 ± 7.4	68.5 ± 10.1	68.5 ± 10.1	0.25
**LNR**					
0 to 0.4	116	94.1 ± 2.3	77.3 ± 5.8	60.6 ± 9.9	
> 0.4	41	86.2 ± 6.6	40.6 ± 13.4	0	**<0.01**
**+LN**					
1 to 3	95	94.3 ± 2.5	76.3 ± 6	58.4 ± 10.5	
> 4	63	89.2 ± 4.7	51.1 ± 13.1	19.2 ± 15.5	**<0.05**
**Stage III**					
IIIA	13	91 ± 8.7	75.8 ± 15.6	75.8 ± 15.6	
IIIB	108	94 ± 2.4	77.1 ± 5.7	56 ± 10.4	
IIIC	42	84.7 ± 7.2	22 ± 17.4	0	**0.01**
**Adjuvant chemotherapy**					
No	42	78.1 ± 6.9	65.6 ± 10.4	43.5 ± 14.4	
Yes	81	97.3 ± 1.9	68.2 ± 7.3	42.2 ± 18.1	0.16
**Gender**					
Female	85	94.4 ± 2.8	66.1 ± 7.9	49.6 ± 11.7	
Male	78	89.7 ± 3.7	73.3 ± 7.8	44 ± 15.2	0.10
**LN resected**					
0 – 11	37	90.9 ± 5	59.9 ± 10.4	59.9 ± 10.4	
≥ 12	123	92.1 ± 2.7	71.7 ± 6.8	40.9 ± 12.3	0.401
**Grade**					
Well-differentiated	6	83.3 ± 15.2	41.7 ± 30.4	–––	
Moderately differentiated	92	93.6 ± 2.8	68.9 ± 7.5	51.9 ± 10.5	
Poorly differentiated	52	90.9 ± 4.4	67 ± 9.9	25.1 ± 19.6	0.61
**Recurrence**					
No	67	83.9 ± 4.7	64.9 ± 6.9	50.5 ± 10.6	
Yes	30	96.6 ± 3.4	64.4 ± 13.1	32.2 ± 17.4	0.41

**Figure 3:**
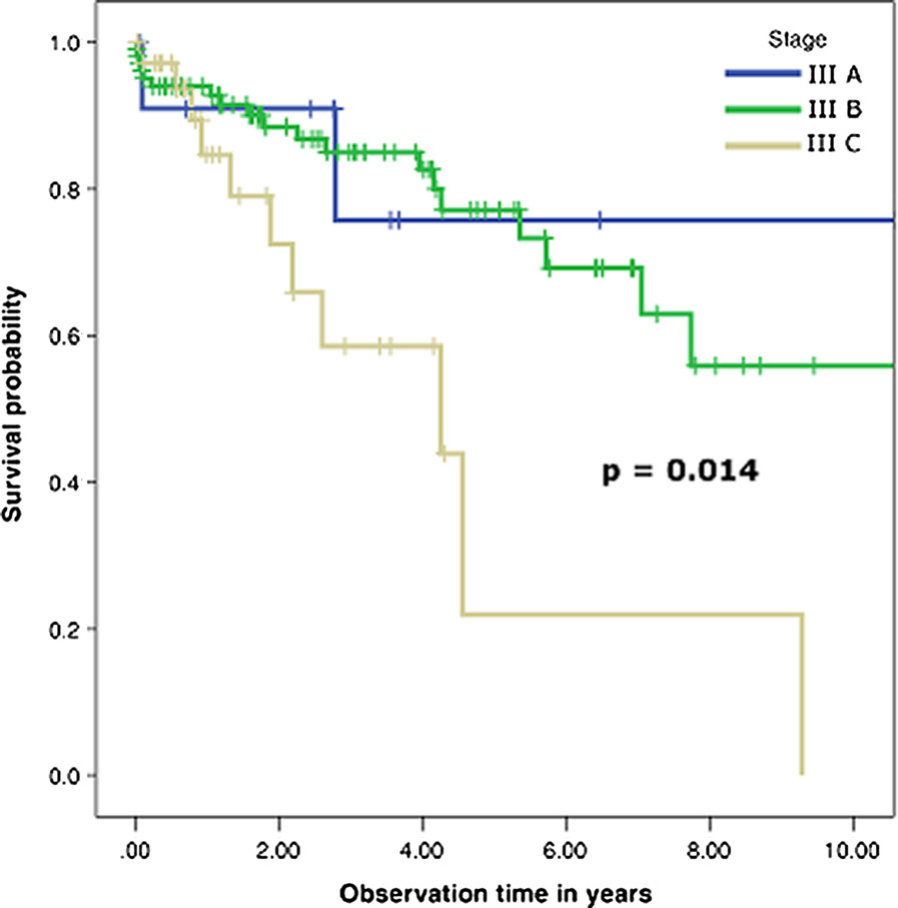
Survival curve for patients with stage III colorectal cancer, stratified as IIIA, IIIB and IIIC.

Survival for patients graded N1 and N2 was 58.4% and 19.2% at 10 years respectively (Table 3, Figure 3).

The total number of LNs resected was not found to be a significant predictor of survival under univariate analysis (*P* = 0.45), but there was a significant proportional correlation between the total number of LNs resected and the number of positive LNs retrieved (*P* < 0.01) (Table 4).

**Table 4:** Relationship of positive LNs (LN) removed to total number of LN resected

**Positive LNs, *n***	**Total LN removed, n (%)**		**Total, n**
	**0 to 11**	**12+**	
1 to 3	27 (28.4)	68 (71.6)	95 (100)_
4+	8 (12.7	55 (87.3)	63 (100)
Total	35	123	158

LNs, lymph nodes; LNR, lymph-node ratio. *P* = 0.002.

**Figure 4:**
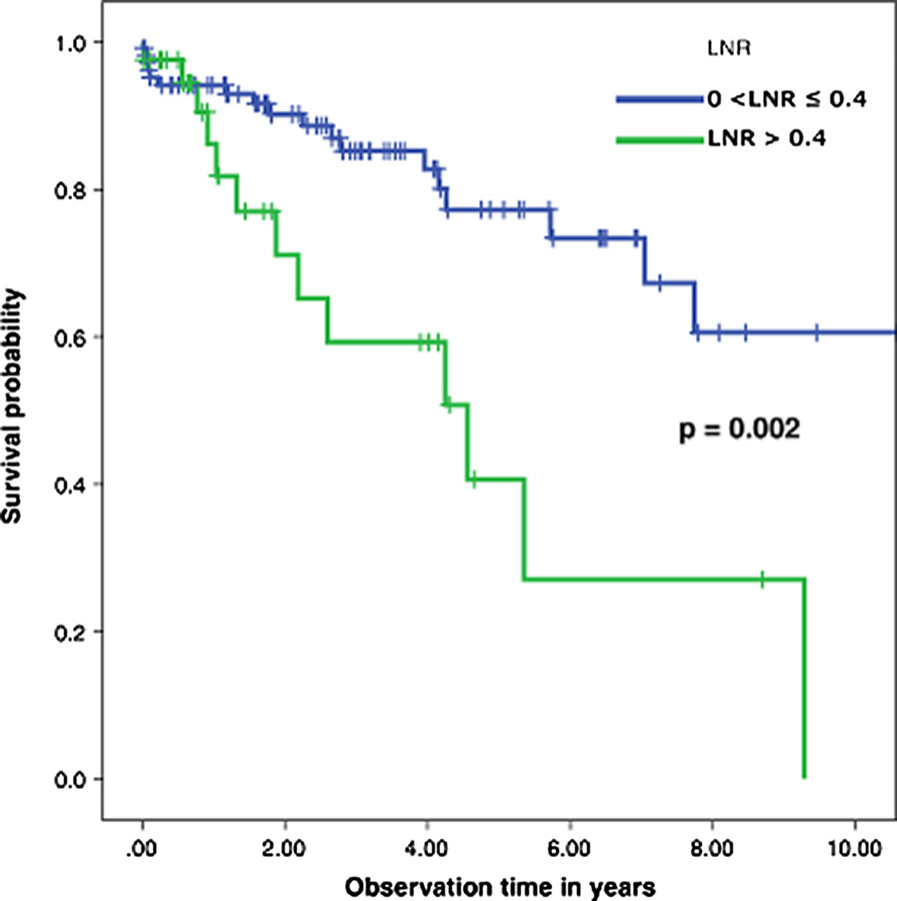
The survival curve for node-positive colorectal cancer patients stratified by lymph-node ratio.

Survival at 1, 5 and 10 years was 94.1%, 77.3% and 60.6%, respectively, for patients with LNR of 0 < to ≤ 0.4, and 86.2%, 40.6% and 0%, respectively, for patients with LNR >4, survival at 1, 5 and 10 years was (Table 3, Figure 4).

### Multivariate analysis

The Cox proportionate hazard model was used to analyze survival rates, and controlled for age, gender, stage III strata (A B, and C), adjuvant therapy, number of positive LNs, and pLNR. LNR > 0.4, controlled for all the other variables, gave HR = 5.25, 95% CI = 1.2 to 22.1. *P* = 0.02 (Table 5).

**Table 5:** Multivariate Cox regression analysis of survival

	**Univariate**			**Multivariate**		
**Characteristics**	**HR**	**95% CI**	***P*-value**	**HR**	**95% CI**	***P*-value**
**Age, years**						
< 50	1	0.385 to 3.051	0.879	1	0.51 to 4.68	0.44
50 – 60	1.084	0.607 to 3.872	0.366	1.54	0.46 to 4.2	0.57
60 – 70	1.533	0.938 to 6.459	0.067	1.39	0.97 to 9.12	0.06
> 70	2.462			2.98		
**Gender**						
Female	1	0.499-2.008	0.998	1	0.39 to 2.25	0.88
Male	1.001			0.93		
**LNR**						
≤ 0.4	1	1.472 to 6.234	**0.003**	1	1.25 to 22.12	**0.02**
> 0.4	3.03			5.25		
**Stage**						
IIIA	1	0.318 to 5.956	0.668	1	0.24 to 5.63	0.86
IIIB	1.377	0.836 to 17.67	0.084	1.15	0.21 to 14.64	0.60
IIIC	3.842			1.76		
**Positive LN**						
1 – 3	1	0.993 to 4.185	0.052	1	0.12 to 2.08	0.35
≥ 4	2.039			0.51		
**Adj chemo**						
No	1	0.279 to 1.235	0.16	1	0.21 to 1.27	0.15
Yes	0.586			0.52		

Adj chemo, adjuvant chemotherapy; LN, lymph node; LNR, lymph-node ratio.

## Discussion

Accurate staging of colorectal cancer is essential for appropriate therapeutic planning. The TNM staging system has taken over from the pathological Duke’s staging system, however it provides limited prognostic information regarding the heterogeneous group of patients with stage III disease. Novel prognostic methods based on three different parameters had been investigated: the total number of LNs collected the number of positive LNs retrieved, and the positive LNR.

The aim of our retrospective study was to compare and re-evaluate different approaches for CRC prognosis in our population of patients with stage III disease.

According to the AJCC [2], stage III CRC is defined by the depth of tumor invasion and the extent of LN involvement in non-metastatic carcinomas. Long-term survival rates depend on and are inversely proportional to the number of LNs involved. Although the TNM system is a reliable therapeutic guide, stage migration, a result of inaccurate TNM staging, has made estimation of future survival rate inconsistent. Furthermore, stage III colorectal cancer is subdivided into A, B, and C according to the number of LNs involved, but this number may vary with the total number of LNs extracted [7,16,17].

The total number of LNs retrieved may be affected by factors such as ages, gender, body mass index, surgical technique and the location of the tumor. Right-sided tumors tend to yield a higher number of retrievable LNs than left-sided tumors [18]. The NCI and RCP both recommend a minimum of 12 nodes should be retrieved [10], but there is no general consensus on the exact number of LNs that must be removed, and surgeons should generally remove as many LNs as possible [11,19]. Taking into consideration all the variables above that could affect the total number of LNs retrieved, our institution has maintained a high resection number over a period of 20 years (mean 20.8, range 2–88) (Table 1).

Reviewing our data, we did not find by univariate analysis a significant correlation between the total number of LNs resected and the survival rate of the patients (*P* = 0.46). This is likely to reflect the high standards of lymphadenectomy, with few patients in this population being understaged. Our findings are consistent with another study conducted using Surveillance, Epidemiology and End Results (SEER) data, which included records from four different hospitals [8].

Because the total number of LNs resected was shown to have poor prognostic value in our population, we investigated the prognostic power of using only the number of positive LNs retrieved for prognostic estimation. This method is currently used for prognostic estimation in the TNM staging system, which classifies colorectal cancer into stage III A, B, or C. According to the AJCC, N denotes the number of positive LNs, and N1 means 1–3 positive LNs collected, while N2 denotes 4 or more positive LNs collected. With regard to this number of positive LN method of prognostication, patients in our population classified as N1 or N2 exhibited approximated 10-year survival rates of 58.4% and 19.2% respectively (*P* = 0.05) (Figure 2). We divided the total number of LNs retrieved into two groups (0–11 and ≥12 LNs removed) and then subdivided each group into two categories (N1 and N2, respectively) (Table 5). Our analysis confirmed that the number of positive LNs retrieved directly correlated with the total number of LNs collected. However, when we used multivariate analysis on the number of positive LNs collected, along with pLNR, tumor stage, and other factors such as age and gender, the number of positive LNs was not found to be significant (*P* = 0.35).

Our results are consistent with an analysis by Moug *et al*. [18], who compared the number of positive LNs and the pLNR in both univariate and multivariate analysis. pLNR maintained its significance as a prognostic factor in both models, whereas the number of positive LNs was not found to be significant when computed along other factors in the multivariate model (Tables 3,4).

To overcome any factors that can affect the yield of LNs, we evaluated a ratio-based classification, the positive LN ratio (pLNR). This ratio takes into account both the total number of LNs retrieved and the actual number of positive LNs found. Because it does not rely on one variable, the pLNR overcomes several limitations pertaining to total LN collection, including surgical and pathological techniques, tumor sites, and even the minimum number of LNs that should be dissected [18]. This method has already been used as a prognostic tool for other tumors such as gastric, pancreatic, and breast [12-15].

Multiple cutoff points for pLNR have been have been presented in the literature. Berger *et al*. used <0.05, 0.05 to 0.19, 0.2 to 0.39 and >0.4 [4]. Our pLNR stratification using 0.4 as the cutoff point is consistent with the work of De Ridder *et al*. [20], who used the same threshold. As expected, our univariate analysis showed that patients with stage IIIA had a better 10-years disease-free survival rate (75.8%) than those with stage IIIB (56%) or stage IIIC (no patients survived) (*P* = 0.01) (Figure 2). It also showed that pLNR had a threshold value of 0.4, with patients having better survival when the ratio was ≤ 0.4 (10-year survival of 60.6%, compared to 0% survival in patients with ratio > 0.4) (*P* < 0.01) (Figure 4).

Using multivariate analysis, we integrated multiple factors to identify which one would be the best prognostic determinant. We looked at age, gender, stage III (stratified into A, B and C), number of positive LNs retrieved, adjuvant chemotherapy, and pLNR. Stage III, although found to be significant in the univariate model, lost its power when computed alongside pLNR. Moreover, pLNR >0.4 proved to have the most significant prognostic factor (HR = 5.25, CI = 1.2 to 22.1, *P* < 0.05), showing that pLNR is indeed an independent prognostic factor for survival in patients with stage III CRC.

A limitation of our is that it was a retrospective review and there was some loss of follow-up for a few few patients. Nonetheless, the results confirm previous studies regarding the prognostic power of the LNR in the colorectal disease. We have to note that our manuscript is the first such study to be conducted in the Middle East.

## Conclusion

Our study confirms the prognostic value of the ratio-based pLNR model to predic survival of patients with stage III CRC relative to stage (AJCC), number of positive LNs, and total LNs retrieved.

## Competing interests

The authors declare that they have no competing interests.

## Authors’ contributions

EE, SA, HH and KM designed the study; EE and HH collected the data; EE drafted the paper; SA supervised the study; and FW and MD edited and corrected the manuscript. DH (PhD statistician) carried the statistical analysis with the help of MO and CM who participated also in doing some parts of the analysis. All authors read and approved the final manuscript.
